# Burnout syndrome and sleep quality in nurses

**DOI:** 10.47626/1679-4435-2021-690

**Published:** 2025-08-25

**Authors:** Maria Gabriela Picagevicz, Joselici da Silva, Francyelle dos Santos Soares, Cristiane Buzanello Donin, Gladson Ricardo Flor Bertolini, Márcia Rosângela Buzanello Azevedo

**Affiliations:** 1 Fisioterapia, Universidade Estadual do Oeste do Paraná, Cascavel, PR, Brazil

**Keywords:** sleep wake-disorders, psychological burnout, dyssomnias, transtornos do sono-vigília, esgotamento psicológico, dissonias

## Abstract

**Introduction:**

Burnout syndrome is complex and causes a great diversity of physical, psychic, and cognitive symptoms.

**Objectives:**

To identify a possible relationship between burnout syndrome and sleep quality of nursing professionals. Methods: Forty-seven nurses working at Hospital Universitário do Oeste do Paraná, from the three shifts, were analyzed. Burnout syndrome was evaluated using the Maslach Burnout Inventory questionnaire, and sleep quality was evaluated using the Pittsburgh Sleep Quality Index. Statistical analysis was performed with the Mann-Whitney Test and the Spearman’s correlation coefficient.

**Results:**

Only three nurses (6.00%) met burnout criteria, but 59.57% had high scores for emotional exhaustion. In relation to sleep quality, eight (17.00%) presented good sleep quality. The correlation between sleep quality and burnout syndrome was significant in the emotional exhaustion dimension (p = 0.0244), being strongly linked to poor sleep quality. There were no differences between working the day and night shifts and the quality of sleep. But when analyzing different sectors, there was a predominance of emergency room + intensive care unit in the presentation of bad dreams or nightmares (p = 0.012).

**Conclusions:**

Sleep alterations were observed in a large portion of the sample, and a high number of individuals presented high levels of emotional exhaustion.

## Abstract (pt)

**Introdução:**

A síndrome de *burnout* é complexa e causa grande diversidade de sintomas físicos, psíquicos e cognitivos.

**Objetivos:**

Identificar possível relação entre a síndrome de *burnout* e qualidade do sono em profissionais da enfermagem.

**Métodos:**

Foram analisados 47 enfermeiros do Hospital Universitário do Oeste do Paraná, trabalhadores dos três turnos. Para avaliação da síndrome de *burnout,* utilizou-se o questionário Maslach Burnout Inventory. Para avaliação da qualidade de sono, utilizou-se o índice de Qualidade do Sono de Pittsburgh. A análise estatística foi realizada com o teste de Mann-Whitney e o coeficiente de correlação de Spearman.

**Resultados:**

Apenas três enfermeiros (6,00%) atenderam aos critérios de *burnout-,* porém, 59,57% apresentaram alta classificação para exaustão emocional. Em relação à qualidade do sono, oito (17,00%) apresentaram boa qualidade do sono. A correlação entre qualidade de sono e síndrome de *burnout* foi significativa na dimensão exaustão emocional (p = 0,0244), sendo fortemente ligada à qualidade de sono ruim. Não houve diferenças entre trabalhar no turno diurno ou noturno e a qualidade do sono. Mas, ao analisar diferentes setores, houve predominância de pronto-socorro + unidade de terapia intensiva na apresentação de sonhos ruins ou pesadelos (p = 0,012).

**Conclusões:**

Foram observadas alterações de sono em boa parte da amostra, e um alto número de indivíduos apresentou altos níveis de exaustão emocional.

## INTRODUCTION

The term “burnout” refers to a syndrome that involves factors associated with exhaustion and strain, representing a response to chronic work-related stressors that cause psychosocial problems affecting the quality of life of professionals of different areas, especially those involving health care, education, and human services.^1,2,3^ It is a multidimensional syndrome characterized by emotional exhaustion related to feelings of fatigue and reduced emotional resources, along with reduced sense of personal accomplishment associated with perceived deterioration of self-competence, and lack of satisfaction with work accomplishments. Finally, burnout syndrome is characterized by depersonalization, which leads to negative attitudes, skepticism, insensitivity, and detachment towards other people. All these factors are concerning and can be detrimental both to the affected individuals and to those under their care.^4^

It is known that burnout syndrome is caused by stress or prolonged work overload, but understanding its initial mechanism remains a challenge to researchers. However, it is known that health care professionals are constantly exposed to many risk factors, the main of which is the very essence of their work – human care –, and their work routine involves pain, distress, and patients’ organic, emotional, and social ill-being. Furthermore, certain forms of work organization give rise to aggravating factors, such as undefined professional roles, work overload, lack of autonomy and authority in decision-making, conflicts between staff members, shift work, large number of shifts, among others, all of which can produce a state of chronic stress.^4,5,6^

Furthermore, the health care sector uses shift work schedules, due to the need to provide 24-hour care. A possible hypothesis is that stress-induced sleep disturbances in the long run may lead to both physical and mental exhaustion. Since disturbed sleep is common in individuals scoring high on burnout, there may be a strong link between stress and sleep, the first being to be the primary cause of persistent psychophysiological insomnia.^7^

In addition to the impact of work on professionals’ physical and mental health, there are implications for the health care sector, because burnout syndrome negatively interferes in the institutional, social, and personal levels. Health care institutions may have as a consequence increased costs resulting from high staff turnover, absenteeism, workers’ health treatments, hiring, and training of new employees. In the social level, professionals with the syndrome may distance themselves from their family, and poorly treated patients need to tackle emotional, physical, and financial problems.^5^

Therefore, additional studies are needed to understand the triggering factors of burnout syndrome to interfere in a preventive manner. This is a complex syndrome that involves an individual process with variations in perceived stress and different psychopathological manifestations. Moreover, it produces a great diversity of physical, psychological and cognitive symptoms, because it includes a myriad of prolonged adaptive responses to stressors, which can jeopardize both individuals and organizations.^1,3^ Therefore, the aim of the present study was to identify a possible correlation between burnout syndrome and sleep quality in nurses.

## METHODS

This is a quantitative, descriptive, cross-sectional study whose target population consisted of nurses working at Hospital Universitário do Oeste do Paraná (HUOP), from the three work shifts. The sample included 47 individuals and was selected by convenience.

The research started after approval by the Human Research Ethics Committee of Universidade Estadual do Oeste do Paraná, under opinion no. 2.200.312. The volunteers have read and signed the Informed Consent Form.

Inclusion criteria were working for at least 1 year at the HUOP and being a permanent employee, whereas exclusion criteria were not answering some of the questions from the data collection tools (Pittsburgh Sleep Quality Index [PSQI] and Maslach Burnout Inventory [MBI]), employees on vacation or absent from work, and pregnant women.

Initially, a sociodemographic and occupational questionnaire was administered. Burnout syndrome was assessed using the MBI questionnaire, developed by Maslach & Jackson,^8^ which is composed of 22 questions and assesses three dimensions of the syndrome (emotional exhaustion, depersonalization, and personal accomplishment), disregarding previous antecedents and consequences of its process. All items are scored on six-point Likert scale ranging from: (0) never, (1) a few times a year, (2) monthly, (3) a few times a month, (4) every week, (5) a few times a week, (6) every day. Question from 1 to 9 identified the level of emotional exhaustion; from 10 to 17, the level of personal accomplishment, and questions from 18 to 22, the level of depersonalization. The values obtained are compared with the reference values of the Center of Advanced Studies on Burnout Syndrome (Núcleo de Estudos Avançados sobre a Síndrome de *Burnout*).^9^ Subsequently, data were analyzed based on the MBI Manual, which uses as a principle for burnout diagnosis a high score for emotional exhaustion and depersonalization and a low score for personal accomplishment. Therefore, the presence of these three criteria in a professional indicates the manifestation of burnout syndrome.

Quality of sleep was evaluated using the PSQI, an instrument validated for the Brazilian population.^10^ This questionnaire analyzes seven components, each presenting an initial score that ranges from 0 to 3 points, and the sum of these components yields a global score from 0 to 21 points, considering that the greater the global score, the worse participants’ quality of sleep. The final values were calculated based on the scoring instructions for the PSQI. The seven components assessed by the instrument are the following: subjective quality of sleep; sleep latency; sleep duration; sleep efficiency; sleep disorders during the past month; use of sleeping medication during the past month; and sleepiness and enthusiasm.^10^

Statistical analysis was performed using the BioEstat software 5.0, considering a 95% confidence level (CI95%) and a level of significance of p < 0.05. The Mann-Whitney test was used for the statistical analysis of the PSQI and MBI questionnaires, with results categorized into daytime and night shifts. The same test was used to assess the sociodemographic questionnaire, categorized into emergency room and intensive care unit (ER + ICU) vs. other departments. The relationship between the PSQI and the MBI was assessed using the Spearmans correlation coefficient. Finally, the chi-square test was used for categorical variables, as well as the independent *t* test.

## RESULTS

Forty-seven nurses working at HUOP participated in this study. There was a predominance of nurses of the female gender, aged 40 years or younger (n = 29; 61.7%), and married or in a common-law marriage.

Table 1 shows the main sociodemographic characteristics of participants, divided into those with good and poor sleep quality. There was a significant difference between the sexes, with women being more likely to have better sleep quality than men (p = 0.036), and between work shifts, with night shift workers reporting worse sleep quality (p = 0.023) than daytime shift workers.

**Table 1:** Patients’ sociodemographic characteristics

Characteristics	Poor sleep quality PSQI > 5 n (%)	Good sleep quality PSQI < 5 n (%)	p-value
Total	36 (76.6)	11 (23.4)	
Sex			
Male	4 (80.0)	1 (20.0)	0.036*
Female	32 (76.2)	10(23.8)	
Marital status			
Married	22 (78.6)	6 (21.4)	0.270
Single	7 (87.5)	1 (12.5)	
Common-law marriage	3 (50.0)	3 (50.0)	
Divorced	3 (75.0)	1 (25.0)	
Widowed	1 (100.0)	0 (0.0)	
Currently studying			
Yes	14 (82.4)	3 (17.6)	0.492
No	22 (73.3)	8 (26.7)	
Department			
ICU + ER	11 (84.6)	2 (15.4)	0.645
Other departments	25 (73.5)	9 (26.5)	
Shift			
Daytime	22 (75.9)	29 (24.1)	0.023*
Night	14 (77.8)	4 (22.2)	

ER = emergency room; ICU = intensive care unit; PSQI = Pittsburgh Sleep

Quality Index.

Chi-square test.

*Statistically significant values at p < 0.05.

**Figure 1:**
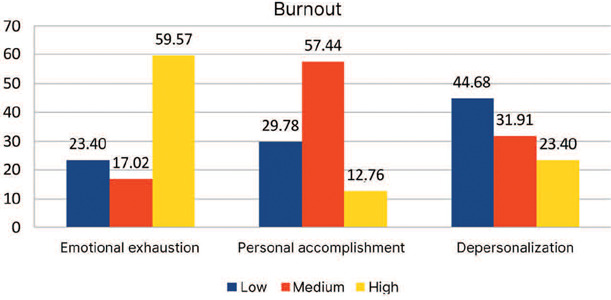
Percentage distribution of the results from the Maslach Burnout Inventory (MBI) questionnaire.

Figure 1 shows the percentage distribution of the results from the MBI questionnaire for the emotional exhaustion, depersonalization, and personal accomplishment dimensions.

It was found that, of the 47 nurses evaluated, only three (6.00%) met burnout criteria. With regard to each burnout dimension, 59.57% of professionals had a high score for emotional exhaustion, 23.40% had a high score for depersonalization, and 29.78% had a low score for professional accomplishment.

In relation to sleep quality (Table 2), of the 47 study participants, only eight (17%) had good sleep quality, whereas the others 39 (82.9%) had poor sleep quality.

**Table 2:** Frequency distribution of Pittsburgh Sleep Quality Index (PSQI) scores

PSQI score	Absolute frequency	Relative frequency
PSQI > 5	39	83
PSQI < 5	8	17
Total	47	100

Score ranges from 0 to 21 points, and values above 5 are considered poor sleep quality.

The correlation between sleep quality and burnout syndrome was statistically significant only for the emotional exhaustion dimension (p = 0.0244), which was strongly related to poor sleep quality in most participants (Table 3).

**Table 3:** Spearman’s correlation coefficient between Pittsburgh Sleep Quality Index (PSQI) and each dimension of burnout syndrome

Emotional exhaustion	Personal accomplishment	Depersonalization
p = 0.0244*	p = 03759	p = 0.1296

* Statistically significant values at p < 0.05.

There was a strong association between emotional exhaustion and poor sleep quality, encompassing situations such as stress, exhaustion, frustration and dissatisfaction at work, and bad nights of sleep. Spearman’s correlation coefficient was used to analyze the intensity and direction of the monotonic relationship between two continuous or ordinal variables (Table 4).

**Table 4:** Spearman’s correlation coefficient between individual variables of the Pittsburgh Sleep Quality Index (PSQI) questionnaire and items of the Maslach Burnout Inventory (MBI)

PSQI	MBI	rho	p-value
Waking up in the middle of the night or early morning	I feel fatigued when I get up in the morning and have to face another day on the job	0.36	0.01*
Overall rating of sleep quality	I feel used up at the end of the workday	0.45	0.00*
Overall rating of sleep quality	I feel like I’m at the end of my rope	0.40	0.00*
Overall rating of sleep quality	I feel fatigued when I get up in the morning and have to face another day on the job	0.33	0.02*
Overall rating of sleep quality	I feel emotionally drained from my work	0.45	0.00*
Overall rating of sleep quality	Working with people directly puts too much stress on me	0.38	0.00*
Overall rating of sleep quality	I feel burned out from my work	0.42	0.00*
Overall rating of sleep quality	I feel I’m working too hard on my job	0.41	0.00*
Overall rating of sleep quality	I feel frustrated by my work	0.38	0.00*
Feeling unwell or lacking enthusiasm for activities of daily living	I feel I’m working too hard at my job	0.51	0.00*
Feeling unwell or lacking enthusiasm for activities of daily living	I feel used up at the end of the workday	0.34	0.01*
Feeling unwell or lacking enthusiasm for activities of daily living	I feel burned out from my work	0.47	0.01*

rho = association value, whose correlation coefficient can range from -1 to +1.

* Statistically significant values at p < 0.05.

There was no statistical significance between working the daytime or night shift and sleep quality. However, when comparing different departments (ER + ICU vs. other departments) in relation to the PSQI, there was a predominance of the first in the occurrence of bad dreams or nightmares (p = 0.012).

Mean scores for sleep quality, as measured by the PSQI, were 8.6+4.4 points on a scale from 0 (better quality) to 21 (worst quality). There was no significant difference (p = 0.097) between work shifts in terms of sleep quality, although there were differences between daytime and night shifts in scores for sleep latency (p = 0.040) and sleep duration (p = 0.033).

Of the overall sample, 59.57% showed high scores for emotional exhaustion, whereas the proportion of high scores was much lower for personal accomplishment and depersonalization (12.76% and 23.40%, respectively) (Table 5).

**Table 5:** Burnout levels classified according to each dimension of the syndrome

Level of burnout	Emotional exhaustion n (%)	Personal accomplishment n (%)	Depersonalization n (%)
Low	11 (23.40)	14 (29.78)	21 (44.68)
Medium	8 (17.02)	27 (57.44)	15 (31.91)
High	28 (59.57)	6 (12.76)	11 (23.40)

## DISCUSSION

The present study found a high percentage of nursing professionals with poor sleep quality a condition that was strongly correlated with emotional exhaustion, which was present in a high proportion of individuals and is considered the initial clinical manifestation of burnout syndrome.^5^ It is worth noting that some authors describe emotional exhaustion as the core of the syndrome, because this symptom elicits and fosters the other ones, with the process advancing in a sequential manner, in which the occurrence of one component of burnout syndrome triggers the next. In addition to emotional exhaustion, as mentioned earlier, the well-defined picture of this syndrome is composed of two other aspects, namely depersonalization and low professional accomplishment.^5,11^

Burnout syndrome has been attributed to several factors, which may be identified as organizational and/or individual factors. Although the relationship between this syndrome and sleep quality has not been clearly defined yet, and further studies are needed to relate these two phenomena in nurses, all findings suggest that one affects the other, thus being closely interconnected in the pattern of physical and emotional exhaustion and fatigue.^12^

Some studies that reinforce this hypothesis showed the relationship between greater sleepiness and worse sleep quality in women scoring high on burnout.^13^ Similarly, Alimoglu & Donmez^12^ observed higher burnout levels in nurses with sleep disorders. High percentages of sleep disturbances were also associated with all three burnout dimensions among nurses, as well as with age and number of night shift per month, confirming their extensive correlation.^14^ Finally, a study with nurses confirmed that the group with burnout had higher insomnia troubles, sleep fragmentation, and non-restorative sleep compared to a healthy control group.^15^ It is suggested that burnout and insomnia, when associated over time, may intensify each other; therefore, either might be a risk factor for the other. There is evidence that untreated exhaustion affects sleep, thus establishing a vicious cycle with a new source of fatigue, leading to its chronicity.^16,17^

Furthermore, an alarming condition, possibly associated with the origin of poor sleep quality among nurses, is work shift. In the present study, night shift work was significantly related to poor sleep quality. A study with similar results showed that most nurses who reported poor sleep quality worked in rotating shifts. Most sociodemographic variables did not affect sleep quality; however, when it comes to burnout components, it was found that high emotional exhaustion and depersonalization were correlated with reduced sleep quality. Nurses who worked in fixed shifts had better sleep quality than those who worked in rotating shifts, suggesting that work shifts were a risk factor for fatigue.^18^ A study conducted with nurses in China confirmed that those who had done shift work were significantly more likely to have poor sleep quality than those who had never done shift work, and that, like in the present study, age, marital status, and having children were not related to sleep quality.^19^

Shift work has a strong influence on manifestations of burnout in nurses and is strongly related to satisfaction at work, possibly making them intend to resign from hospital or even abandon their career. Some findings confirm the greater prevalence of burnout among shift workers compared with those who did not work shifts; moreover, longer sleeping hours per day were associated with lower odds of burnout among shift workers.^20^

Nursing is a health care area that uses work shift schedules, but professionals who work the night shift are more exposed to sleep disorders and diseases, because their working conditions often lead to accumulated sleep debt, reducing its quantity and quality, and generating chronic sleep deprivation. Therefore, professionals of this shift become more vulnerable to work-related fatigue and thus present with excessive daytime sleepiness, which causes multiple health problems in the long term.

Results from several studies on the same issue show that night shift nurses have worse subjective sleep quality, daytime dysfunction, and sleep disturbances compared to daytime shift nurses. This may be explained because night shifts lead to sleep deprivation at night, thus disturbing circadian rhythms and directly interfering with individuals’ physical and mental performance, which in most cases causes emotional, physical, social, and professional losses.^21,22^ Additionally, it should be considered that the activities performed by these professionals at sites like the ICU may produce physical discomfort, with repercussions on social life and performance at work^23^; moreover, nursing professionals are constantly exposed to stressful factors in their work environment, such as lack of human resources and professional autonomy.^6^

It is understood that the temporal organization of night work is undeniably detrimental to workers’ health, not only in the physical aspect but also in the psychic, emotional, and social aspects. By reversing the sleep-wake cycle because of night work, i.e., sleeping during the day and working at night, workers induce an internal desynchronization of biological and circadian rhythms, in addition to favoring conflicts in their social lives. Maladaptation to shift work, especially night work, produces physical manifestations such as insomnia or excessive daytime sleepiness, chronic fatigue, and stress, all caused by a sudden change in work schedule or by continuously working in a fixed or rotating schedule, when there is a rupture of mechanisms involved in circadian and homeostatic regulation of sleep, which are altered by different sleeping times. If the same work shift is maintained for a long time, some professionals adapt themselves to this work schedule, whereas others never do.^24^

It bears noting that the limitation of the study was its small sample size, because, although the study hospital is a regional referral center and has a large nursing staff, many nurses refused to participate, alleging lack of time to answer the questionnaires; furthermore, the presence of sleep problems prior to the beginning of the study was not investigated. Moreover, it is worth highlighting that convenience sampling also limits study findings, because the psychological factors related to the items analyzed may have justified the refusal of several professionals in participating in the study which should not be considered unusual, since many professionals neglect even precautionary measures to prevent occupational accidents.^25^

## CONCLUSIONS

The sample was characterized by sleep alterations, especially among night shift workers, and, despite the small number of individuals with burnout, a great number of participants presented high levels of emotional exhaustion.
